# A Preliminary Review of Fatigue Among Rail Staff

**DOI:** 10.3389/fpsyg.2018.00634

**Published:** 2018-05-07

**Authors:** Jialin Fan, Andrew P. Smith

**Affiliations:** Centre for Occupational and Health Psychology, School of Psychology, Cardiff University, Cardiff, United Kingdom

**Keywords:** fatigue, rail industry, work demands, railway incidents, countermeasure, preliminary review

## Abstract

**Background:** Fatigue is a severe problem in the rail industry, which may jeopardize train crew's health and safety. Nonetheless, a preliminary review of all empirical evidence for train crew fatigue is still lacking. The aim of the present paper is, therefore, to provide a preliminary description of occupational fatigue in the rail industry. This paper reviews the literature with the research question examining the risk factors associated with train crew fatigue, covering both papers published in refereed journals and reports from trade organizations and regulators. It assesses the progress of research on railway fatigue, including research on the main risk factors for railway fatigue, the association between fatigue and railway incidents, and how to better manage fatigue in the railway industry.

**Methods:** Systematic searches were performed in both science and industry databases. The searches considered studies published before August 2017. The main exclusion criterion was fatigue not being directly measured through subjective or objective methods.

**Results:** A total of 31 studies were included in the main review. The causes of fatigue included long working hours, heavy workload, early morning or night shifts, and insufficient sleep. Poor working environment, particular job roles, and individual differences also contributed to fatigue.

**Conclusion:** Fatigue in the rail industry includes most of the features of occupational fatigue, and it is also subject to industry-specific factors. The effect of fatigue on well-being and the fatigued population in the railway industry are still not clear. Future studies can consider associations between occupational risk factors and perceived fatigue by examining the prevalence of fatigue and identifying the potential risk factors in staff within the railway industry.

## Introduction

The railway system of the United Kingdom is the oldest in the world. From steam pioneers through the railway entrepreneurial boom, to a loss-generating nationalized British Rail, then to the privatization of railway operations, the history of the UK rail industry has ridden a technological and social wave for nearly 200 years. Since privatization, the number of rail passengers has grown rapidly. The public image of rail travel, however, was damaged by some prominent accidents shortly after privatization. These accidents included the Southall rail crash and the Ladbroke Grove rail crash, which both resulted in deaths and hundreds of injuries, as well as the Hatfield accident, which exposed major stewardship shortcomings (British Office of Rail Regulation, 2006). These serious human error-related accidents led to reforms in railway management and safety.

Rail crew fatigue is not only a problem in the UK, it can be a problem in all those countries having railway transport. The majority of the job roles in train crew are safety-critical, such as being a train driver, engineer, signaller (i.e., controller), conductor (i.e., guard), and even a station worker. Although some of these job roles do not involve the actual operation of the train, they are responsible for operational and safety duties. For example, the conductors ensure that the train stays on schedule, deal with unexpected delays or emergencies, and ensure that the train follows applicable safety rules to avoid any incident. Station employees carry out duties at the station, which include not only selling and checking tickets, but also making sure that passengers get on and off the train safely, and signaling the conductors or driver to depart.

Failure to manage fatigue among the train crew may increase the risk to employees' health and train safety. The term *fatigue* is synonymous with a generalized stress response over time (Cameron, 1973), and it is similar to conditions like burnout (Huibers et al., 2003). There are different stages of fatigue, including acute fatigue and chronic fatigue. For example, fatigue that occurs during or after work is known as *acute fatigue*, while the fatigue carried forward over days is known as *chronic fatigue*. According to the Oxford Dictionary 2013, fatigue in humans is “extreme tiredness arising from mental or physical effort.” The subjective feelings of fatigue include descriptors such as tired, lacking energy, sleepy, or exhausted (Job and Dalziel, 2001; Shen et al., 2006). Generally, fatigue results in the deterioration of attention, perception, decision-making, and skilled performance (Cercarelli and Ryan, 1996; Beurskens et al., 2000), or a physiological state characterized by a decreased response of cells, tissues, or organs after excessive stress or activity (Hirshkowitz, 2013). In an occupational context, fatigue may occur during or after work (i.e., acute fatigue), or before work when a person has not fully recovered from previous fatigue through the normal periods of rest and sleep before the onset of the next set of demands (i.e., chronic fatigue; Cameron, 1973). The causes of occupational fatigue are varied, including generic causes not specific to the workplace (e.g., sleep loss, time of day), and work-related causes (e.g., job demands, work duration, and job control); it is also affected by individual differences. In research on occupational fatigue, workload is often equated to job demands, which may contribute to the development of fatigue and related reductions in performance. Fatigue resulting in the deterioration of attention and impaired performance in the workplace, brings danger to those working in safety-critical job roles.

**Figure 1:**
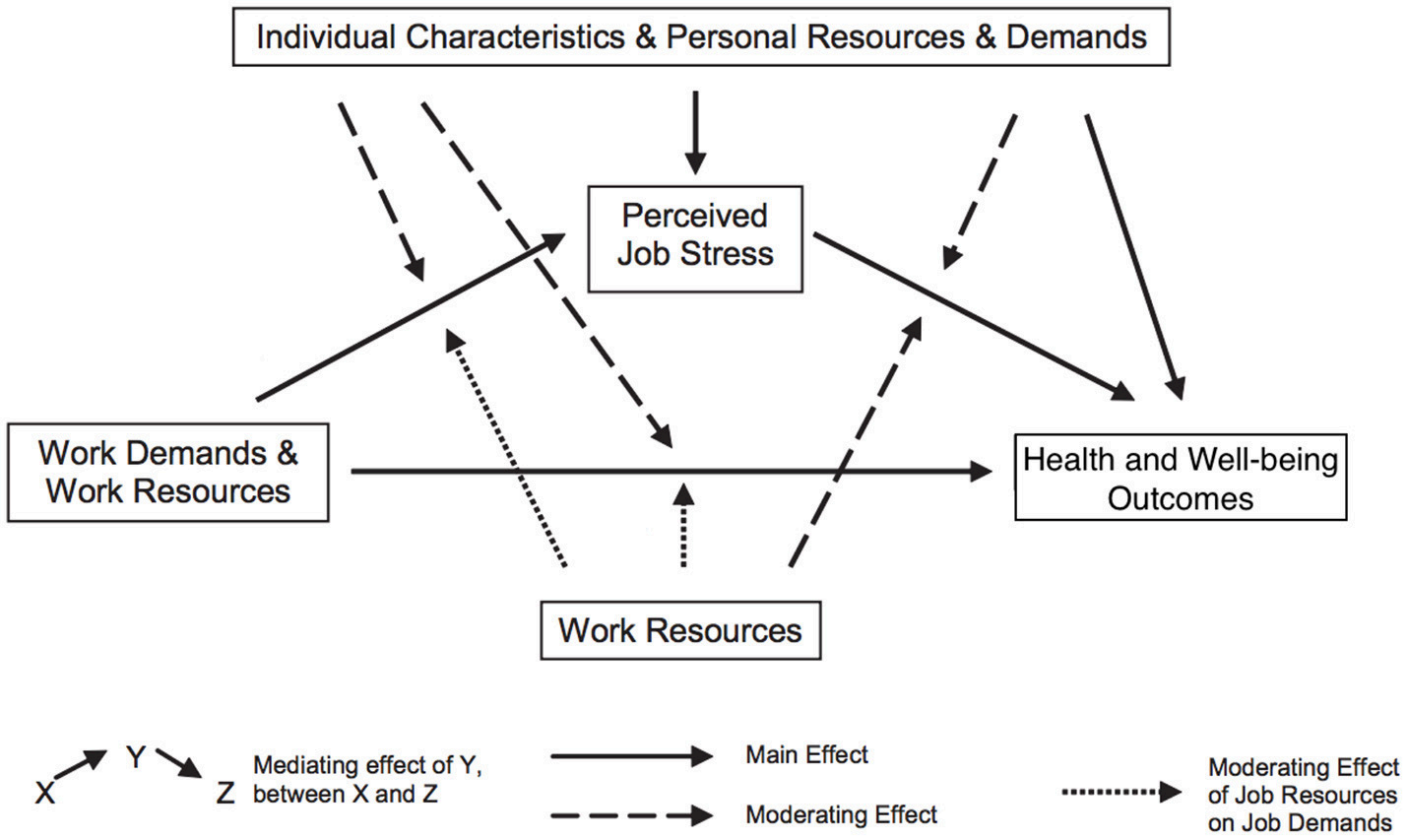
DRIVE model.

In present review, the Demands, Resources, and Individual Effects (DRIVE) model (Mark and Smith, 2008) was used as the framework for assessing fatigue (Figure 1). It was initially a stress model but has also been used in occupational fatigue studies. This model demonstrates the important role of work demands, work resources (i.e., support and control), and individual differences in influencing perceived job stress (i.e., fatigue) and well-being outcomes. It proposed that the subjective appraisal of fatigue could mediate the relationship between the environment and the outcomes. A recent study (Fan and Smith, in press) has found such a mediating effect of fatigue. Although this model also suggested that individual differences may moderate the relationships between environment, fatigue and outcomes, this a moderating effect was not found in subsequent studies (e.g., Capasso et al., 2016).

Fatigue is a severe problem in the transport sectors, including road, sea, air and rail. Smith (2007) reviewed fatigue in these transport sectors. This research indicated that the different transport sectors have similar fatigue-related problems and the scientific approach to fatigue used to define general principles should apply to all these sectors. However, Smith also suggested that a “one size fits all” approach to regulation may be inappropriate to all, as there are different features between industries. Phillips (2014) reviewed research on fatigue in operators working on road, sea, and rail. His review found that although the features of the transport sector influenced the focus of studies, there was good coverage of the effects of both psychosocial work factors (e.g., workload, control support) and working time on sleep and fatigue. Also, the outcomes of fatigue in transport sectors are self-reported well-being, general health, shift-work disorder, mood, and objective psychomotor performance. In the rail industry especially, poor work-life balance and sickness absence are considered to be the outcomes of fatigue.

Just like other workers, train crew are exposed to general work characteristics associated with fatigue. They are also subject to industry-specific factors potentially related to fatigue. For example, harsh working environments, tasks requiring sustained vigilance, and shift-work systems have been associated with fatigue (Lal and Craig, 2001; British Office of Rail Regulation, 2012). Since automation technology has been applied in the workplace, work in the railway industry imposes more cognitive demands while physical demands have diminished (Young et al., 2015). The jobs requiring sustained vigilance in the modern rail transport may result in heavy mental workload and increased fatigue. Moreover, fatigue is considered to be a causal factor in train accident and incident reports (British Rail Safety Standards Board, 2005; British Rail Accident Investigation Branch, 2008, 2010). Recently, fatigue and its impact on safety-critical performance have been suggested as a key issue in the rail industry (Bowler and Gibbon, 2015); however, thus far, no systematic attempt to determine levels of staff fatigue in the rail industry, and the associated risk factors has been made.

In order to address fatigue in the rail industry, it is important to first place the research questions in context by systematically reviewing the existing literature. The present article aims to provide a preliminary description of the literature on fatigue in the rail sector. It is intended to cover both papers published in refereed journals and reports from trade organizations and regulators. In light of past studies, the features of rail crew fatigue and mechanisms for measuring the effect of fatigue on performance are suggested as search areas.

## Methods

The main search engines used for literature searches were PubMed, Google Scholar, and Scopus. The search terms used were “railway fatigue,” “rail fatigue,” “train staff fatigue,” and “train driver/controller/conductor fatigue.” References within the resulting papers were also checked for useful research.

**Figure 2:**
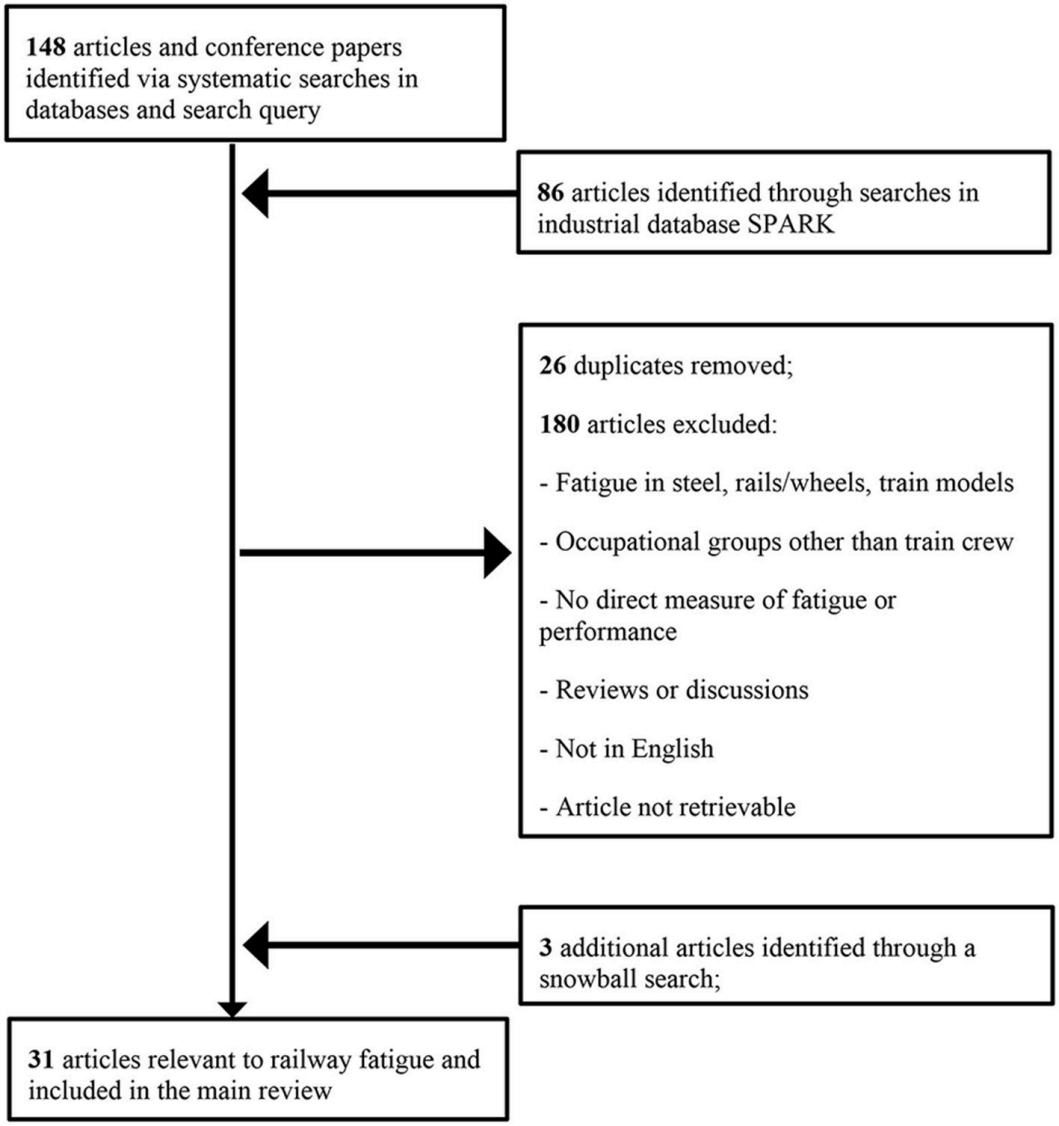
Flow chart illustrating the process of selection of articles for main body of literature review.

The papers reviewed in this article described original research concerning the stressors of fatigue and the effects of fatigue on performance in the railway industry. Studies were considered eligible if (a) participants were members of the train crew, (b) research questions involved the factors associated with train crew fatigue, (c) fatigue was assessed through subjective ratings of fatigue or its synonyms (e.g., tiredness or alertness), or through objective measures of fatigue or performance, and (d) research articles provided data. Duplicate articles and research that primarily concerned ergonomic factors, train models, and biological indicators of fatigue (e.g., heart rate) were excluded. The numbers of papers excluded and included are summarized in Figure 2.

Historically, the field of rail fatigue research has been smaller than that of other transport groups; thus, there is very little relevant literature on train crew fatigue and its countermeasures. For example, a search of “railway fatigue” via Google Scholar, there are 84 results in total, only one of which is actually related to the current study. SPARK, a database for the railway industry sector incorporating the Rail Safety and Standards Board (RSSB) Human Factors library, was used, therefore, for searching further related literature. In addition, 13 government or organization documents published on the websites of the United Kingdom's ORR, RAIB, and RSSB, the Swedish National Road and Transport Research Institute, and the Japanese Railway Technical Research Institute are related to this study and will also be reviewed.

## Results

As shown in Figure 1, 148 papers from science databases and 86 papers from the industrial database SPARK were identified through systematic searches. Based on full-text reading, 31 studies were included in the main review and these articles are marked with an asterisk in the reference list. The main exclusion criterion was fatigue not being measured through subjective or objective methods. Supplementary Table 1 (in the supplementary material) shows the details of the reviewed studies. The sample size of these studies varied from *n* = 9 in a field study with continuous rest time and vigilance performance measured over 3 days, to *n* = 1,758, in a large-scale cross-sectional online questionnaire. Sixty-five percent of the studies were based on large samples (i.e., sample size equal or larger than 50). Train drivers were the most commonly examined group, followed by engineers and controllers (i.e., dispatcher or signalman). Five studies compared more than two job role groups. The most common focus in terms of risk factors for fatigue were the working time factor (65%; *n* = 20) and the working demands factor (61%; *n* = 19), followed by the sleep and rest factor, working environment factor, and individual differences. As for fatigue measurement, 17 studies used subjective measures, three studies used objective measures, and the remaining studies used both.

### Risk factors for railway fatigue

Fatigue is difficult to define, with many different and complex symptoms in different jobs, but the British Office of Rail Regulation (2012) defines railway fatigue as a state of “perceived weariness that can result from prolonged working, heavy workload, insufficient rest, and inadequate sleep” (p. 6). This definition implicates potential causes of fatigue and makes the distinction between task-related and sleep-related fatigue. Task-related fatigue usually reflects the workload of the task being carried out, working hours, and shift-work, while the sleep-related fatigue is affected by sleep loss and insufficient rest.

In earlier research, Pollard (1990) explored the risk factors of different working patterns for train drivers, particularly those factors which might contribute to fatigue. The main causes of fatigue that interviewees frequently mentioned were long working times, heavy workload, shift-work, and poor working environments. In addition, long commute times, uncertainty of on-call jobs, and conflicts with other job roles were reported to be potential stressors causing fatigue. In later studies, such risk factors for fatigue were identified in different job roles of train crew (e.g., controllers; Gertler and Nash, 2004). The risk factors described in the following sections are working hours, workload, timing of work (i.e., shift-work), job type and environment, lifestyle and other individual factors, sleep and rest.

#### Working hours

Seventeen studies reported the effects of work demand factors on fatigue. Among these studies, nine longitudinal/process studies investigated the length of work time, with seven focusing on train drivers (McGuffog et al., 2004; Darwent et al., 2008; Dorrian et al., 2008; Prakash et al., 2011; Cabonl et al., 2012; Robertson et al., 2013; Kazemi et al., 2016), and two on controllers (Popkin et al., 2001; Korunka et al., 2012). Overall, no matter whether in passenger or freight train operating companies, the train drivers working longer hours had higher fatigue scores than those working fewer hours. Darwent et al. (2008) stated that significant cumulative fatigue and sleep loss appeared throughout the duration of driving. Drivers were, however, able to sustain vigilant performance during driving despite having incurred a significant sleep debt. Kazemi et al. (2016) suggested that train drivers on long-haul trips usually had longer rest periods between the outward trip and return, which could compensate for the side effects of long working times. The results of the fatigue studies on controllers were similar to those on train drivers.

#### Workload

Workload was examined in 12 studies, with five cross-sectional mail surveys (Prakash et al., 2011; Zoer et al., 2011; Cotrim et al., 2017; Fan and Smith, 2017; Tsao et al., 2017) and eight longitudinal studies (Popkin et al., 2001; Roach et al., 2001; McGuffog et al., 2004; Dorrian et al., 2007b, 2008, 2011; De Luca et al., 2009; Dunn and Williamson, 2012). These studies all showed positive associations between workload and fatigue either in train drivers or in other train crew members. Tsao et al. (2017) found that workload and overtime work led to fatigue in both drivers and engineers, while Fan and Smith (2017) found that high workload resulted in higher subjective fatigue across the train crew. A study of train drivers (Dorrian et al., 2007b) showed that with a high workload, high levels of fatigue resulted in cognitive disengagement from the driving task, leading to a dramatic increase in accident risk. Zoer et al. (2011) noted that the high workload in train crew (especially in the younger crew members) was associated with higher levels of fatigue, as well as higher risk of mental health complaints. De Luca et al. (2009) explained that the physiological effort required to remain a necessary level of alertness and performance under monotonous conditions results in oxidative stress which indicated fatigue.

#### Timing of work

Twenty-three studies investigated the effect of time into the work period and the differences between night shifts and day shifts. Among these, six were cross-sectional mail surveys (Kibblewhite, 2003; Ku and Smith, 2010; Zoer et al., 2011; Zimmermann et al., 2015; Cotrim et al., 2017; Fan and Smith, 2017), and 17 were longitudinal/process studies (Popkin et al., 2001; Roach et al., 2001; Harma et al., 2002; McGuffog et al., 2004; Dorrian et al., 2006, 2007a, 2008, 2011; Darwent et al., 2008, 2015; Jay et al., 2008; Cabonl et al., 2012; Korunka et al., 2012; Paterson et al., 2012; Cebola et al., 2013; de Araújo Fernandes et al., 2013; Robertson et al., 2013). Most of these studies showed that night shifts result in fatigue (e.g., Dorrian et al., 2011), as well as sleepiness and cumulative sleep loss (Darwent et al., 2008; Cotrim et al., 2017). First, Popkin et al. (2001) observed that fatigue developed more quickly during night shifts than during day and evening shifts. Then, Harma et al. (2002) found that in both night shifts and early morning shifts, fatigue and severe sleepiness at work were very common. Darwent et al. (2015) suggested that fatigue during the shifts was mainly affected by amounts of rest and sleep before work. Korunka et al. (2012), however, suggested that fatigue during the shift was not only affected by recovery during break phases before work, but also by fatigue at shift onset and perceived workload during the shift.

#### Job type and environment

Generally, most of the existing research investigated fatigue in train drivers; however, train drivers are not representative of the entire train crew. In this review, 15 studies sampled different job roles in the rail industry, including railway controller, conductor, engineer, or station worker (Popkin et al., 2001; Roach et al., 2001; Harma et al., 2002; Sherry and Philbrick, 2004; Ku and Smith, 2010; Dorrian et al., 2011; Prakash et al., 2011; Zoer et al., 2011; Korunka et al., 2012; Paterson et al., 2012; Cebola et al., 2013; ärmä et al., 2014; Zimmermann et al., 2015; Cotrim et al., 2017; Fan and Smith, 2017; Tsao et al., 2017).

Three studies focused on fatigue in railway controllers (Popkin et al., 2001; Korunka et al., 2012; Cotrim et al., 2017), two in engineers (Roach et al., 2001; Cebola et al., 2013) and one in conductors (ärmä et al., 2014). The results of these studies showed a high prevalence of fatigue in these job roles during night shifts. In addition, fatigue caused the train engineers to disengage from work, and there was a trade-off between safety and efficiency (Roach et al., 2001), particularly for those who were working on-call (Cebola et al., 2013). ärmä et al. (2014) studied fatigue in conductors and noted that the conductors were exposed to very high levels of noise, which could be above the recommendation of the World Health Organization (WHO). Such noise could adversely affect working performance, cause intolerance or distraction, and result in poor health outcomes (e.g., fatigue, tinnitus).

Another 10 studies compared two or more job roles in the railway industry. Differences in workload, work hours (i.e., length of work, the percentage of night shifts, and the number of consecutive shifts), and sleep loss were found across different job roles (Harma et al., 2002; Dorrian et al., 2011), and were consistent with the nature of each role. For example, the engineer crew worked a high percentage of night shifts because most train maintenance and rail repairs were scheduled at night to avoid daytime traffic and allow trains to be used in the day. Additionally, environmental factors such as noise level in the workplace seemed to appear in particular job roles and affect fatigue (Prakash et al., 2011; ärmä et al., 2014). For instance, noise and vibration had more impact on conductors and drivers and were associated with their fatigue, while fumes were more likely to affect the engineers but were not found to contribute to their fatigue (Fan and Smith, 2017).

#### Lifestyle and other individual factors

Five studies investigated individual differences, with three investigating lifestyle (Roach et al., 2001; Paterson et al., 2012; Fan and Smith, 2017), one age (Zoer et al., 2011), and one chronotypes (de Araújo Fernandes et al., 2013). Fan and Smith (2017) found that train crew members with an unhealthy lifestyle or negative personality were more likely to report high fatigue. The other two studies involving lifestyle suggested that smoking and drinking alcohol were related to performance impairment, while no effect of caffeine consumption was found. Smokers reported lower subjective sleep quality, which could increase fatigue-related risk. The impairment in performance and safety due to fatigue was in a range similar to that associated with the levels of alcohol consumption (Roach et al., 2001). Zoer et al. (2011) noted that heavier emotional and mental workloads in the younger staff members and lack of social support for older staff members were associated with fatigue and ill health. de Araújo Fernandes et al. (2013) stated that evening chronotypes remained awake for a longer time before the night shift and had worse life quality compared to morning types. However, there was no significant difference in fatigue and performance between these two chronotypes.

#### Sleep and rest

Twelve studies reported the effect of sleep and rest on fatigue. Sleep and rest variables commonly studied were usually collected using standard self-report measures and included sleep length, sleep quality, rest time during work, and frequency of rest (Jay et al., 2008; Dorrian et al., 2011; Prakash et al., 2011; Cabonl et al., 2012; Cebola et al., 2013; Robertson et al., 2013; Zimmermann et al., 2015; Tsao et al., 2017). Sleep quantity and quality were also collected objectively in several studies using actigraphs (Sherry and Philbrick, 2004; Dorrian et al., 2007a, 2011; Paterson et al., 2012; Darwent et al., 2015). These studies supported a view that sufficient sleep and rest helps the train crew recover from fatigue. Also, the prophylactic napping before starting shift-work helps crew members cope with fatigue (Jay et al., 2008; Darwent et al., 2015). Sleep deprivation which is influenced by shift-work, results in fatigue and sleepiness at work (Cabonl et al., 2012). Darwent et al. (2015) found that higher levels of fatigue were generally associated with significant reductions in the amount of sleep obtained before shifts, despite the individual differences in fatigue resistance (e.g., smoking or not, different chronotypes).

### Fatigue measurement in these studies

Thirty studies used subjective measures, objective measures (mainly the Psychomotor Vigilance Test; PVT), or both. There was one study which used biological measurement of oxidative stress as an indicator of fatigue (De Luca et al., 2009). Seventeen studies only used subjective fatigue measures, including visual analog scale (VAS), Samn–Perelli Fatigue Checklist, Job Stress Rating Scale (JSRS), and other self-assessments (Harma et al., 2002; Kibblewhite, 2003; McGuffog et al., 2004; Ku and Smith, 2010; Dorrian et al., 2011; Prakash et al., 2011; Zoer et al., 2011; Cabonl et al., 2012; Dunn and Williamson, 2012; Korunka et al., 2012; Paterson et al., 2012; Cebola et al., 2013; Robertson et al., 2013; ärmä et al., 2014; Zimmermann et al., 2015; Kazemi et al., 2016; Cotrim et al., 2017; Tsao et al., 2017). In contrast, three studies used only objective fatigue measures, including the PVT (Darwent et al., 2008) and the Fatigue Audit InterDyne (FAID; Dorrian et al., 2007a; Darwent et al., 2015). The rest of the studies used both kinds of measures (Popkin et al., 2001; Roach et al., 2001; Sherry and Philbrick, 2004; Dorrian et al., 2006, 2007b, 2008; Jay et al., 2008; Dunn and Williamson, 2012; de Araújo Fernandes et al., 2013). The subjective fatigue measures were suitable for diary studies, where train crew could report their acute fatigue levels before, during, and after a shift (Harma et al., 2002; McGuffog et al., 2004; Jay et al., 2008; Dorrian et al., 2011; Korunka et al., 2012; Paterson et al., 2012; Cebola et al., 2013; Robertson et al., 2013). Dorrian et al. (2008) compared simulated driving, the PVT, and subjective ratings. They found that the self-ratings were more strongly associated with PVT performance than the “real world” tasks.

### Fatigue in railway accident or incident investigations

There were 98 rail investigation reports found in the SPARK database, 23 of which identified fatigue as one of the contributory causes of the train incident or accident. Two Japanese reviews (Kogi and Ohta, 1975; Ugajin, 1999) state that the human error in railway accidents was associated with drowsiness, motivation, and time of day, which might also be related to fatigue. In Buck and Lamonde's (1993) review, evidence supported such relationships between critical railway accidents and train crew fatigue, as well as such factors as time of day, shift-work, and work-sleep-rest cycles. Recently, reviews of British rail incidents confirmed that fatigue was a cause in about 21% of the sampled high-risk railway incidents, in which fatigue mainly resulted from negative work-life balance, insufficient sleep, shift pattern design, and the control of working length (Gibson et al., 2015; Gibson, 2016).

These views were supported by an exploratory study of UK rail workers' perceptions of accident risk factors (Morgan et al., 2016). This study demonstrated the impact of shift-work, commuting time, work-life balance, and time pressure on perceived stress and fatigue at work. Moreover, decision-making and risk-management abilities were challenged and impaired by fatigue and the job demands under time pressure, resulting in increased risks of error, accidents, and incidents, and the increased likelihood of near-miss occurrences and underreporting. Dorrian et al. (2007a) observed that train operators with a higher risk of fatigue had more frequent speed violations and heavier brake use on flat sections of the route, both of which would increase the safety risk. In addition, time of day was found to affect fatigue and increased both the non-fatal and fatal injury risks of train crew during night-time work (Calabrese et al., 2017). Particularly for the roadway workers (i.e., engineers and conductors), night time work was more hazardous than daytime work.

### Fatigue prediction systems and countermeasures in the railway

The Driver's Safety Device is a basic safety protection system in most trains to prevent train catastrophes should the driver become incapacitated (e.g., fall asleep, lose consciousness). It is also commonly called the “dead man's handle” or “dead man's pedal.” When this safety device is not held in place by the driver, the brake will be activated. If the driver ignores audible and visual warnings that they should be taking appropriate action, automatic braking systems will be activated to stop the train (Phillips and Sagberg, 2014). Despite such devices, fatigue is still a serious risk to railway safety. Fatigue also presents dangers other than those related to sleepiness, such as inattention or poor decision-making (Phillips and Sagberg, 2014). Considering that drivers often have the power to override automatic systems, the mentally fatigued driver may be as much a risk as a sleepy driver to railway safety. Besides, the automatic braking system works only when the driver is fatigued already, and is not adequate for addressing other train crew members' fatigue (e.g., controllers). Detecting and managing the train crew's fatigue in advance, therefore, is another strategy for safety protection.

Current fatigue detection by prediction systems in the railway industry can be classified into four categories (reviewed by Anund et al., 2015). The first group of systems is based on eye detection. This group of systems usually uses infrared cameras and measures eye blinks, gaze, and pupil size, but false alarms still occur. The second group of systems is based on physical activity, but is still being developed. The third group is part of the prediction system developed by the transport machine industry (e.g., the Automatic Train Control and Automatic Train Protection system). The final group of systems uses multiple measuring approaches and combines different types of sensors. The understanding of fatigue prevention and management, however, is hampered by a lack of the instruments needed to measure fatigue.

The UK Health and Safety Executive (HSE) has its own fatigue prediction tool called the Fatigue and Risk Index (British Health and Safety Executive, 2006). It was designed primarily to assess and compare the risks from fatigue associated with rotating shift patterns, but it can also be used to identify any particular shift, within a given schedule, that may be of concern. It calculates one fatigue index and one risk index based on cumulative fatigue, workload, alertness, shift length, time of day, commuting time, frequency and length of breaks, and the recovery from a sequence of shifts. It is important to note, however, that this assessment is limited, as it does not consider individual differences (e.g., lifestyle, age) or specific work-related issues (e.g., exposure to noise or vibration). The job role might also affect the risk of fatigue, but the mathematical formulae used in this assessment could not account for such variations.

The main coping strategies in the rail industry are breaks, napping, and caffeine use (British Rail Safety Standards and Board, 2012). Breaks are an effective way of controlling the build-up of fatigue. The finding of TRAIN, a Swedish research project, suggests that workers should take a 12-h break between shifts to avoid serious fatigue problems (Kecklund et al., 2001). Fatigue should be compensated with recovery and rest, not with economic compensation. Meanwhile, the Driving and Rest Time Hours in International Rail Transport Act (2008, p. 475) suggested taking a minimum 45-min rest after every 4.5-h working period. Shifts longer than 12 h lead to fatigue and increase the risk of accidents, and fatigue builds cumulatively with every successive shift when breaks in-between are insufficient (Anderson et al., 2013). Although it is difficult to develop prescriptive rules that balance security and operational effectiveness efficiently at the organizational level covering the entire rail industry, it is important to build a framework of fatigue management that prescribes hours of work and rest, especially for shifts that last more than 12 h. The train companies could use fatigue modeling tools to improve shift-work arrangements (British Health and Safety Executive, 2006; British Rail Safety Standards Board, 2016a). British Office of Rail Regulation (2011) recommended the use of a comprehensive sleep disorder management tool and promote the tool for fatigue management.

Napping is an effective countermeasure to address task-related fatigue. (British Rail Safety Standards Board, 2005) found that napping was used as a coping strategy by one-third of drivers, especially prior to night shifts. Caffeinated drinks were used as a fatigue countermeasure by half of the train drivers in the RSSB survey (2005), and around 5% used caffeine tablets. The employees were informed about the adverse effects of caffeine as well as its benefits, together with advice to use it only when needed at work, as the body gets used to caffeine use and consequently, its effects are reduced. Armed with this information, the drivers would be able to choose whether to use caffeine as a fatigue countermeasure.

The strategy behind the use of these two countermeasures (i.e., napping and caffeine use), and evaluation of them, are not commonly seen in the literature. In addition, the safety bodies of the UK rail industry published several guidelines for train companies' use in managing fatigue and for staff members' use to self-check and deal with fatigue problems (e.g., British Rail Safety Standards and Board, 2012; British Rail Safety Standards Board, 2016b,c).

## Discussion

### Summary of main findings

Occupational fatigue is generally caused by workload, lack of control and support, working time, and individual differences, and it leads to performance impairment and ill health. Fatigue in the rail industry shows most of the features of occupational fatigue, and is also subject to industry-specific factors. Previous research had indicated that railway fatigue was associated with workload, working time, shift-work, sleep and rest, and health-related behaviors. These risk factors for fatigue, however, seem to differ between job roles in the railway due to the nature of the duties, and the differences between job roles are still unclear. Similarly, it is unclear if environmental factors affect fatigue, or if different job roles with different workloads result in different levels of perceived fatigue. Although the effect of fatigue on safety and health has been observed in government reports (British Rail Accident Investigation Branch, 2008; British Office of Rail Regulation, 2011; British Rail Safety Standards and Board, 2014), the evidence on the effects of fatigue on well-being and cognitive performance is less clear in the studies reviewed. Ku and Smith (2010) suggested that fatigue problems are associated with poor social well-being and more health complaints among train conductors and engineers, but there is still a lack of studies covering most of the other job roles.

Most of the existing studies used subjective fatigue ratings or both subjective fatigue ratings and the PVT to assess fatigue, suggesting that in future studies of railway fatigue, fatigue self-assessment and PVT will also likely be used. Although the PVT was broadly used as an objective indicator of fatigue, it is not clear how subjective fatigue is associated with PVT outcomes. Also, the current version of PVT is a portable testing device, but it is costly to use with large samples, which is a motivator for developing a lightweight and more convenient version of PVT (e.g., an online version of PVT). In addition, the diaries have been used to track and assess the changes in fatigue levels before, during and after a shift. Future studies could also try to combine cognitive performance tests with a fatigue diary.

Fatigue has gained attention in the railway industry, as it was one of the main contributing factors in human error-related rail accidents and incidents. Several fatigue management tools and systems have already been developed for use. However, it is commonly noted that there is a lack of systematic evaluations of whether these tools actually reduce fatigue (Anund et al., 2015). The main difficulty is monitoring and detecting fatigue in a timely manner, which would then allow the fatigue management tools to provide support to the fatigued train crew.

### Comparison to other transport sectors

As Smith (2007) suggested, the fatigue problems in rail transport are similar to those in other transport sectors. The risk factors for fatigue in rail include long working hours, heavy workload, shift-work, and insufficient sleep and rest, which also predict fatigue in other industries. Zoer et al. (2011) noted that compared with elder crew members, younger staff with a high workload were more likely to report higher levels of fatigue, and a greater risk of mental health complaints. The potential reason for this is because of the culture of the apprenticeship system in railway industry, where younger member may have less voice in choosing personal-preferred work patterns and be more likely to have the heavier workload.

The Driver's Safety Device on trains is similar to those warning systems equipped on aircraft which is used to alert the pilot if the aircraft is in immediate danger (e.g., flying into the ground or having a collision with another aircraft). The shipping industry also has a similar system, the Vessel Traffic Service (VTS), which continuously monitors all ships to ensure the watch-keepers are alert and the ships are on the planned trip with no deviation.

Caffeine and napping are the common and main countermeasures of fatigue for the individual in all these sectors. However, napping during work is allowed in aviation, while staff should stay awake and alert in rail and other sectors. Compared with other transport workers, flight crew often have better rest policies and rest environments (Gregory et al., 2010). On some long-haul flights, pilots even have a room for rest with beds inside. Drivers in road transport often use short breaks during a journey to recover from fatigue, which involves stopping to take a short walk, while train drivers usually do not have enough time stopped at one station to have such a break.

### Limitations

Due to the scarcity of relevant literature on train crew fatigue, the present systematic review might be limited in its conclusions by the samples, parameters, and fatigue measurements in the studies. Moreover, very few studies are comprehensive in the inclusion of most of the risk factors of fatigue and all job roles of the train crew.

## Conclusions

Previous research has indicated that high work demand, length of work, and shift-work cause railway fatigue. Individual differences, differences between job roles, and environmental factors may also be involved in the variation in fatigue, but currently there is a lack of evidence showing clear associations between these factors. In particular, very few studies have covered most of the job roles in the railway industry. The effect of fatigue on well-being and the fatigued population in the railway industry are still not clear.

Future research on train crew fatigue should consider associations between occupational risk factors and perceived fatigue by examining the prevalence of fatigue and identifying the potential risk factors in staff from the railway industry. The research should also build a detailed picture of the relationships between workplace stressors, individual differences, fatigue, and well-being outcomes, covering all job roles in the railway industry. It should cover the fatigue-related issues raised in railway accident reports and provide empirical support for potential organizational interventions to combat fatigue.

## Author contributions

AS formulated the research question and designed the study. JF conducted the analyses, interpreted the data and drafted the original manuscript. AS then revised the manuscript for important intellectual content, and both the authors approved the final version for publication. Both the authors agreed to be held accountable for all aspects of the work in ensuring that questions related to accuracy and integrity are appropriately investigated and resolved.

### Conflict of interest statement

The authors declare that the research was conducted in the absence of any commercial or financial relationships that could be construed as a potential conflict of interest.
