# Supplementary material for: A Preliminary Review of Fatigue Among Rail Staff
Source: Front Psychol. 2018 May 7;9:634. doi: 10.3389/fpsyg.2018.00634 (PMC5949530; doi:10.3389/fpsyg.2018.00634)
Supplement: Supplementary file 1 [file Table_1.DOCX]

Supplementary Material

A Preliminary Review of Fatigue among Rail Staff

Jialin Fan, Andrew P. Smith^*^

*** Correspondence:** Andrew P. Smith: SmithAP@Cardiff.ac.uk

# Supplementary Tables

**Supplementary Table 1.** Characteristics of Reviewed Studies about Risk Factors of Fatigue in the Railway

| **Reference** | **Sample Size and Characteristics** | **Study Purpose** | **Fatigue Measurement** | **Risk Factors** | **Fatigue-Related Outcome** |
| --- | --- | --- | --- | --- | --- |
| Cabonl et al. (2012) | Survey: N =565  Field Study: N = 25  Train Driver  In France | Evaluation of the impact of hours of work on fatigue. | Survey: Fatigue & Shift-work Questionnaire  Field study: Karolinska Sleepiness Scale (KSS) | Working demand factor: working hour  Working time factor: time to work  Sleep & rest factor: sleepiness, sleep loss, sleep quality | A large amount of sleep deprivation associated with some duty hours (night and morning) which result in fatigue. Sleep loss is significantly increased when working periods have more than 5 to 6 duties. Subjective sleepiness is at a moderate level before the trips and increases after the trips. Fatigue should be managed both at organisational and individual level. |
| Cebola et al. (2013) | N = 24  Engineer (Fleet)  In the U.K. | Investigation of the particular relationship between on-call work, shift work, fatigue, anxiety performance, and mood. | 5-point rating scales  Diary | Working time factor: uncertainty on-call job, shift work  Sleep & rest factor: poor quality of sleep | The results show that on-call work leads to an increase of anxiety, affects the sleep quality of on-call workers, and leads to increased fatigue when compared to not on-call work. |
| Cotrim et al. (2017) | N = 97 (all males)  Railway controllers  Age: M = 44.8  Seniority M = 21.7, with 50% of the sample worked for more than 13 years  In Portugal | Investigation of the influence of work and individual determinants in sleepiness. | 5-point rating scales in the Questionnaire REQUEST  Copenhagen Psychosocial Questionnaire II (COPSOQ II) | Working demand factor: job demand  Working time factor: night shift  Psychosocial work factors: job satisfaction | The main predictors of sleepiness were job demands, job satisfaction, and night shift fatigue.  High prevalence of fatigue during the night shift.  High levels of dissatisfaction with shift system may have influenced fatigue perception. |
| Darwent et al. (2008). | N = 10 (all males)  Train driver  Age: mean(SD) = 43.30 (±7.42)  In Australia | Examination of the sleep and vigilance performance of train drivers during an extended 106-hr relay operation (included a 16-hr layover). | Hand-held psychomotor vigilance task (PVT) | Working demand factor: long time work  Working time factor: shift-work | Significant cumulative sleep loss appeared across duration of the operation.  Drivers sustained vigilant performance for the duration of operation despite significant sleep debt. |
| Darwent et al. (2015). | N = 322 (309 males, 15 females)  Train driver  Age: 39.5 (±14.2)  Been shift workers for 19.3 (±9.0) years  In Australia | Development of sleep transfer functions describing the likely distributions of sleep around fatigue level. | Fatigue Audit InterDyne (FAID) | Working time factor: shift-work  (6:00 am to 1:59 pm, 2:00 pm to 9:59 pm, and 10:00 pm to 5:59 am.)  Sleep history | Higher fatigue score categories were associated with significant reductions of sleep obtained before shifts. Only minor differences in prior sleep amounts were observed between morning, afternoon, and night shifts. |
| de Araujo Fernandes et al. (2013) | N = 91 (all males)  Train driver  In Brazil | Comparison of the sleep pattern, fatigue and life quality between different chronotypes in train drivers. | Visual analogue scale; Subjective questionnaire  Psychomotor Vigilance Task (PVT) | Working time factor: shift-work, time into work period (four days)  Individual difference: chronotypes | Evening types remained awake for a longer time before the night shift and had worse life quality compared to morning types. No significant difference of fatigue and PVT performance between different chronotypes. |
| de Luca et al. (2009) | Total N = 136  Rail group N = 63 (60 males, 3 females)  Train engine drivers  Age: 26–53  Professional seniority (years): 3 – 30 | Studying the biochemical features of oxidative and neurological stress in the blood and urine of three selected groups of professionals at high health risk (e.g., train engine drivers, pilots, and cosmonauts) to prove the working hypothesis that a relevant molecular basis for their fatigue and professional stress-related health disorders could be the impaired equilibrium between oxidant/antioxidant levels in the organism. | Lipophilic/hydrophilic low-molecular weight antioxidant (AO) and AO enzyme activities  Nitric oxide, superoxide anion, hydroperoxide production  Urinary catecholamine/ serotonine metabolites and lipoperoxidation markers | Working demand factor: the physiological effort required to regain a level of alertness which allows adequate performance under monotonous conditions | The Rail group displayed a significant AO depletion, with severely depleted plasma levels of vitamin C, accounting for inadequate diet regimen. Detoxification mechanisms were also impaired in the whole group. This group are subjected to alteration of sleep schedules and circadian rhythm and, most importantly, to monotony stress, which is a consequence of the physiological effort required to regain a level of alertness which allows adequate performance under monotonous conditions, necessary to avoid train malfunction and life-threatening accidents. The consequent psychoemotional wear, connected with the responsibility for the life of passengers, is among the main causes of frequent occurrence of burnout syndrome and immunological dysfunction. To control the health hazards it is strongly recommended the supplementation of chemopreventive agents, to allow the restoration of adequate AO defenses. |
| Dorrian et al. (2007a) | N = 50 (all male)  Age: 24–56  Train driver | Investigation of the effects of fatigue on train driving using data loggers. | Fatigue Audit Interdyne (FAID) | Working time factor: shift work  Rest and sleep factor: rest time | Fatigued driving becomes less well-planned, resulting in reduced efficiency (e.g. increased fuel consumption) and safety (e.g. braking and speeding violations). Fatigue may manifest differentially depending on track grade. In certain areas, fatigue causes increased fuel use and economic cost, and in others, reduced safety through driving violations. |
| Dorrian et al. (2006) | N = 20 (all males)  Train driver  Age: 39.4 ± 9.4  In Australia | Investigation of the relationship between fatigue, braking behaviour and speeding during four speed-restricted areas on a simulated train track. | 10-min PVT  VAS alertness rating | Working time factor: shift-work (10:00 to 18:00, 23:00 to 07:00)  Outcomes: more errors, less brake, higher speed | As expected, increases in fatigue produced increases in frequency and duration of attentional lapses. Translating PVT lapse durations into operational terms, a train driver will have travelled between 25 m and 125 m during the lapse period. Clearly, this could have a significant effect on the ability to plan and negotiate a speed restriction adequately, and as a consequence, pose a serious safety risk. |
| Dorrian et al. (2007b) | N = 20 (all males)  Train driver  In Australia | Investigation of the effects of sleep loss and fatigue on performance in a rail simulator. | PVT  Self-rated performance  Visual Analogue Scales: alertness | Working demand factor: workload (number of penalty brake applications) | High levels of fatigue result in a cognitive disengagement from the driving task, lead to a dramatic increase in accident risk. Ratings were more accurate for PVT performance than for the “real world” task. |
| Dorrian et al. (2008) | N = 15 (9 males, 6 females)  Train crew  In Australia | Examination of the validity and reliability of using the EDA to assess elevated levels of sleepiness and reduced alertness. | Samn–Perelli Fatigue Checklist  Psychomotor vigilance (PVT) | Working hour factor: time into work period (3 days)  Work demands factor: 28 h of sustained wakefulness | Simulated driving, PVT, and subjective ratings indicated increasing sleepiness and fatigue during the experimental period. The electrodermal activity (EDA) indicator did not sense increased sleepiness and fatigue at levels produced in the present study. |
| Dorrian et al. (2011) | N = 90  Driver, controller, guard, resurface crew, signaller, terminal operator  In Australia | Investigation of sleep, work hours, workload, and fatigue in a series of field studies involving a wide variety of job types in the railway industry, including drivers, train controllers, guards, resurface crews, signallers, and terminal operators. | Samn–Perelli Fatigue Scale  Actigraphs | Working demand factor: workload, working hours  Working time factor: shift work  Sleep & rest factor: sleep hours  Other: job role | Sleep length, wakefulness and work hours, workload significantly influences fatigue. Fatigue at work is likely to be as prevalent for other job roles (e.g., signallers) as it is for drivers.  Differences in work hours (shift length, percentage night shifts and number of consecutive shifts), sleep/wake cycle were found across different job roles. For example, the resurfacing crew worked a high percentage of night shifts because most track repairs are scheduled at night to avoid daytime traffic. Differences in reported workload across job roles were clear and consistent with the nature of each role. |
| Dunn and Williamson (2012) | N = 58 (all males)  Train driver  In Australia | Examination of the effect of cognitive demand on train drivers' driving performance on monotonous routes | Visual analogue scale, Driving Fatigue Questionnaire (slightly modified for train driving rather than car driving) | Work demand factor: cognitive demands | There were seriously detrimental effects of the combination of monotony and low task demands on fatigue and performance.  A relatively minor increase in cognitive demand can mitigate adverse monotony-related effects on performance for extended periods of time. |
| Fan and Smith (2017) | N = 1067  Conductors, drivers, station workers, engineers, administrators, managers, at-seat catering stewards, and controllers. | A large-scale survey investigating whether workload (high job demands) was associated with fatigue. | Smith Well-being Questionnaire (SWELL), derived from the Well-being Process Questionnaire (WPQ) | Work demand factor: job demand  Working time factor: shift work  Job resources: job support and control, organisational factors  Working environment factor: exposure to noise and vibration  Individual difference factor: personality, health-related lifestyle | High workload, poor job control and support, shift-work, exposure to noise and vibration, unhealthy lifestyle and negative personality would result in fatigue. As one of the several predictors of subjective fatigue, high workload results in higher levels of subjective fatigue. Meanwhile, both high workload and high levels of subjective fatigue were found to correlate with poor subjective reports of performance efficiency. |
| Hamidi et al. (2014) | N = 167 (all males)  Conductor  Age: 29.3 ± 3.2  In Iran | Investigation of noise among train conductors and its consequences. | Self-assessment of fatigue | Working environment factor: noise | Conductors’ noise exposure level was very high and much above the recommendation of the World Health Organization (WHO).  Noise adversely affects work performance and cause intolerance or distraction. High level of noise and noise annoyance among train conductors resulted in the poor health outcomes (e.g. fatigue, sleeplessness, tinnitus) of the conductors. |
| Harma et al. (2002) | N = 230 (all males)  60.4% train driver, 51.1% controllers  In Finland | Examination of the prevalence of severe sleepiness at work in train drivers and controllers. The effect of different shift and sleep history on the risk of severe sleepiness at work. | 5-point Visual analogue scale  Diary | Working time factor: shift work (i.e., early morning shift, day shift, evening shift, night shift), time to work | Fatigue and severe sleepiness at work are very common among train drivers and controllers, especially during night shift and early morning shift.  Shift timing, shift length, and off-duty time, in addition to actions aiming at extending the main sleep period, would probably decrease severe sleepiness in railway transportation. |
| Jay et al. (2008) | N = 9 (all males)  Train driver  In Australia | Investigation of the impact of a shift system on drivers' fatigue and recovery in 3 days following each trip. | Samn–Perelli Fatigue Checklist  Psychomotor vigilance task (PVT) | Working time factor: 8h-8h-off  Sleep & rest factor: rest time | While there was a clear trend for fatigue levels to be elevated at the end of each working shift, each 8-hour rest period appeared sufficient to reduce fatigue to levels recorded prior to departure. |
| Kazemi et al. (2016) | N = 97 (all males)  Train driver  In Iran | Comparison of train drivers’ fatigue and workload between long-haul and short-haul trips. | 7-point Samn-Perelli Fatigue Scale | Working demands factor: long/short work hours | Fatigue reached a high level at the end of work. Fatigue and workload were not very different in both long shifts and short shifts. |
| Kibblewhit (2003) | N = 15 (14 males, 1 female)  Driver in Train Operating Company (TOC) | Investigation of the risk factors of fatigue-related inattention and distraction for train safety. | Self-report fatigue  45-min interview | Working time factor: shift work, shift length  Job resources: communication, job control  Work environment factor: cab temperature | Shift work was identified as an aspect of the role and the consequent fatigue which accrued, leading to potential inattention. Heat, fatigue, and monotony are factors that may reduce the driver’s ability to maintain active cognitive control. |
| Korunka et al. (2012) | N = 626  Controller  In Europe | Investigation of the role of recovery and detachment in the break period between two shifts for fatigue. | Diary study: self-assessment of the current level of fatigue | Work demand factor: long work times (12 hr day shift-24 hr off- 12 hr night shift, 24 hr off-12 hr night shift, 48 hr off-12 hr day shift)  Working time factor: time to work | Fatigue during the shift was not only affected by recovery and psychological detachment during break phases before work but also by fatigue at shift onset and perceived workload during the shift. Workload affects fatigue in the day shift but fades away in the night shift. Both psychological detachment and recovery are important off-the-job inhibitors of fatigue after 4-hour shifts but not 8- or 12-hour shifts. |
| Ku and Smith (2010) | N = 125 (124 males, 1 female)  45.9% engineers, 54.1% conductors  In United States | Examination of the job-related factors on fatigue, health, and social well-being | Four questions about fatigue: Sleep Quality, Anxiety and two POMS factors (Fatigue – Inertia, Vigour – Activity) | Working time factor: work scheduling  Job resources: social support, organisational factors | Social support is an important mediator between scheduling and fatigue.  Organisational factors and scheduling items had the same underlying structures and could be combined into one factor. |
| McGuffog et al. (2004) | Questionnaire:  N = 460  Diary Study: N = 22  Train driver  In the UK | Investigation of the fatigue-related risk of current shift patterns and the strategies for risk reduction and control. | Questionnaire  28-day diary | Working demand factor: mental workload, cumulative duty hours, without break  Working time factor: shift work (early morning shift), time of day, commuting time  Work environment factor: noise | The main issue that has been identified relating to fatigue and accident risk was shift-work. Early starts are a feature of railway operation and may be associated with particular problems when effects due to the restriction of sleep prior to duty are exacerbated by high work rates in the morning hours. Another issue is the role of rest days in limiting the build-up in fatigue after a long sequence of consecutive shifts. |
| Paterson et al. (2012) | N = 40 (37 males, 3 females)  23 train driver  17 other (engineer, shunters, and team leaders)  Age: 45.1 (±12.5) | Investigation of the sleep behaviour of shift workers to identify employees who are more likely to be impaired by fatigue. | Samn–Perelli fatigue scale  Work diary (2 weeks)  Actigraphs | Working time factor: shift work  Individual difference: parent or not, smoking or not  Sleep & rest factor: sleep quantity and quality | Differences in work type and workload may influence sleep.  Participants with dependents were found to obtain significantly less sleep than participants without dependents. Smokers reported lower subjective sleep quality, independent of sleep type (i.e., day or night). |
| Prakash et al. (2011) | N = 200  50% health train driver, 50% controller (a sedentary job) | Identification of job-related factors in the railway and environment stressors of railway engine pilots’ fatigue and their level of occupational stress. | Job Stress Rating Scale (JSRS) | Work demand factor: workload/ long duties  Rest & sleep factor: improper rest  Working environment factor: noise, vibration  Other: job role | The study reveals that Railway Pilots have high levels of job stress while controllers have mild levels of job stress.  Job stress has been found to significantly correlate with stressors like vibration, noise, long duties, improper rest, sleep disturbances, irregular food habits, and fatigue. |
| Popkin et al. (2001) | N=37  Controller  20 from freight, 17 from passenger train  In United States | Examination of sources and levels of railroad controller's workload, stress, and fatigue. | Subjective rating  14-day sleep log  Actigraphy (sleep/awake cycle) | Working demand factor: overwork  Working time factor: Shift work (especially night shifts) | The results identified that shift work contributes to fatigue. Fatigue for both freight and passenger operation despatchers accumulated more quickly during night shift than day and evening shifts. |
| Roach et al. (2001) | N = 20 (all male)  Engineer  Age: 39.4 (±9.4)  In Australia | Quantify the effects of fatigue on performance in a simulated work environment (the rail simulator) and compare them with the effects of alcohol consumption. | Psychomotor vigilance (PVT)  Self-assessments of alertness and performance | Individual difference factor: alcohol  Working time factor: Night shift  Work demand factor: workload (two or three consecutive night shifts) | Fatigue caused participants to disengage from operating the simulator so that the safety was traded off against efficiency.  The neurobehavioural performance impairment due to fatigue was similar to that associated with moderate levels of alcohol consumption. |
| Robertson et al. (2013) | N = 102  Freight train driver  Mean age: 46 | Investigation of the factors which contribute to the onset of fatigue. Evaluation of fatigue effect on work, accidents, and incidents. | Samn–Perelli Fatigue Scale  Diary | Working demand factor: length of work  Working time factor: time of day, shift work  Sleep & rest factor: the extent of the recovery | Freight drivers often experience long periods of inactivity waiting for track access and long periods without a break, plus requirements to work at night. There are also last-minute extensions to the shift duration due to delays. These factors have the potential to contribute, singly or in combination, to levels of fatigue and the overall risk of accidents. |
| Sherry and Philbrick (2004) | N=21  Engineer | Evaluation of the functionality of improving individual fatigue management in railroad engineer with actigraph feedback. | Self-assessment (Denver Fatigue Adjective Checklist, Stanford Sleepiness Scale, etc.)  30-day sleep log,  Actigraphy | Rest and sleep factor: sleep activity, work/rest habits | The readings (or the feedback) of performance on actigraphs made participants more aware of their fatigue levels to a considerable or great degree and increased their awareness of the need for rest. |
| Tsao et al. (2017) | N = 524  297 engineers (male = 282, female = 15)  227 drivers (all male, 95.2% from freight)  In China | Investigation of fatigue prevalence in Chinese railway employees, and the influential factors of their fatigue. | Self-assessment (MFI-20, NASA-TLX, questionnaire of fatigue-related factors) | Working demand factor: workload, overtime work  Working environment factor:  Physical working environment (noise, vibration, light, temperature, comfort)  Job resources: job control  work/rest rhythm | For the locomotive employees, higher workload and working overtime led to fatigue. For the rail maintenance department, workload and work/rest rhythm directly influenced fatigue, while workload was influenced by work/rest rhythm and the intensity of overtime work.  They suggest that managers in the railway system should arrange an appropriate work/rest schedule, and improve the physical working environment for the operators to manage the fatigue of railway employees. |
| Zimmermann et al. (2015) | N = 1758  Train crew  In Canada | Investigation of the overall picture of the fatigue status in the Canadian on-call freight rail staff. | Subjective fatigue rating (online) | Work demand factor: long working hours  Working time factor: unpredictable schedule (earlier shift), on-call job task.  Sleep & rest factor: sleep, opportunities for rest | Train crews routinely operate while fatigued, which remains a safety issue. Fatigue is normalized as part of the job and not taken seriously as a safety issue at various management levels. Such cultural issues and the production goals are usually prioritised over safety. The conflict of interest created by remuneration schemes may pose obstacles to progress on fatigue risk management. |
| Zoer et al. (2011). | N = 827  278 Train driver  193 Conductor  193 Station worker  155 Service electricians (engineer) | Exploration of the associations between aspects of psychosocial workload and mental health complaints in four age groups of railway workers. | Questionnaire on the Experience and Evaluation of Work (QEEW; Van Veldhoven et al. 2002) | Individual differences: age (22-35, 36-45, 46-55, 55+)  Work demand factor: workload  Other: job type | Worse emotional and mental workload in the younger employees and lack of social support in older employees were associated with fatigue and related to a higher risk of having mental health complaints. |
